# Highly accurate sigmoidal fitting of real-time PCR data by introducing a parameter for asymmetry

**DOI:** 10.1186/1471-2105-9-221

**Published:** 2008-04-29

**Authors:** Andrej-Nikolai Spiess, Caroline Feig, Christian Ritz

**Affiliations:** 1 Department of Andrology, University Hospital Hamburg-Eppendorf, Hamburg, Germany; 2 Statistics Group, Department of Natural Sciences, Faculty of Life Sciences, University of Copenhagen, Denmark

## Abstract

**Background:**

Fitting four-parameter sigmoidal models is one of the methods established in the analysis of quantitative real-time PCR (qPCR) data. We had observed that these models are not optimal in the fitting outcome due to the inherent constraint of symmetry around the point of inflection. Thus, we found it necessary to employ a mathematical algorithm that circumvents this problem and which utilizes an additional parameter for accommodating asymmetrical structures in sigmoidal qPCR data.

**Results:**

The four-parameter models were compared to their five-parameter counterparts by means of nested F-tests based on the residual variance, thus acquiring a statistical measure for higher performance. For nearly all qPCR data we examined, five-parameter models resulted in a significantly better fit. Furthermore, accuracy and precision for the estimation of efficiencies and calculation of quantitative ratios were assessed with four independent dilution datasets and compared to the most commonly used quantification methods. It could be shown that the five-parameter model exhibits an accuracy and precision more similar to the non-sigmoidal quantification methods.

**Conclusion:**

The five-parameter sigmoidal models outperform the established four-parameter model with high statistical significance. The estimation of essential PCR parameters such as PCR efficiency, threshold cycles and initial template fluorescence is more robust and has smaller variance. The model is implemented in the *qpcR *package for the freely available statistical *R *environment. The package can be downloaded from the author's homepage.

## Background

Quantitative real-time polymerase chain reaction (qPCR) has become an invaluable tool for monitoring gene expression changes, combining the sensitivity of the PCR technique with the ability to quantify transcriptional changes with high accuracy [1]. Several different methods exist in respect to hardware (i.e. cappillary-based systems or thermal block-based systems) or fluorescence chemistry and design. Using the DNA intercalating dye SYBR Green I is one of most widely applied systems, as the fluorescence readout can be obtained from any PCR amplicon irrespective of its sequence. This way qPCR experiments can be conducted fast and with many different sequences, as is the case in screening and evaluating differential gene expression obtained from microarray experiments [2,3].

When investigating differential gene expression, qPCR data of two or more different conditions (such as control/treatment or healthy/pathological) are compared by using the fluorescence data acquired by the hardware. One approach is the comparison of the threshold cycles, when the fluorescence of the qPCR reaction rises significantly above the background level, commonly done by the ΔΔCt methods. Originally developed with the tenet that the PCR efficiency is 2 [4], this was soon extended by the long known observation that PCR efficiency can have smaller values and be very different between two different amplicons, as is the case when normalizing a gene of interest against a 'housekeeping' gene. This necessitates the calculation of the efficiency in order to derive a realistic estimate of the expression changes. Various algorithms have been developed such as estimation from the slope of a calibration curve [5,6] or from a linear fit of the logarithmized data within the exponential region either defined by the 'midpoint' [7] or the region with highest linearity ('window-of-linearity')[8].

In contrast to the above described linear quantitation methods, sigmoidal models have been developed for non-linear fitting of the PCR data, most commonly the *Boltzmann *or logistic sigmoidal function [9,10,17]. The advantage of non-linear fitting is the paradigm that PCR efficiency is not a constant but a variable that changes during PCR, having a maximum in the exponential phase of the reaction and declining in later cycles of the reaction when reagents get depleted, thus leading to the sigmoidal curvature. Non-linear fitting can then be used to calculate threshold fluorescence, cycle-dependent efficiency (E_cyc_) and estimation of the starting template amount (F_0_). The described sigmoidal qPCR models are four-parameter models that define by their fitted function the parameters ground asymptote, slope, point of inflection and maximum asymptote. The fitted parameters of logistic curves describe the qPCR data usually well and supersede other models like *Gompertz *and *Chapman *[11].

Although PCR data can be fitted with the four-parameter approach, this model implies symmetry of the lower and upper part of the curve, which results in the same curvature on either side of the inflection point. We found that this poses some essential problems that needed to be solved. Firstly, it is not evident that qPCR curves can be assumed to be symmetric. That this is indeed not the fact will be shown in this work. Secondly, and most important for the quantification aspect, is that fitting four-parameter models with symmetry as an inherent constraint onto asymmetric data will consequently lead to suboptimal fits and estimation of parameters [12].

We investigated the effect of applying logistic and also log-logistic five-parameter models to qPCR data, in which the fifth parameter takes a possible asymmetrical structure of the data into account. Five-parameter models have only just recently found their way into the dose-response analysis of immunological data [13].

Furthermore, we tested the significance of this approach with various statistical measures by comparing to fits of models with less parameters. The here described algorithms are implemented (besides many other functions) in the *qpcR *library [22] extension for the open source statistical programming environment *R *[23].

## Results

### The f-parameter in different PCR regimes

**Figure 1:**
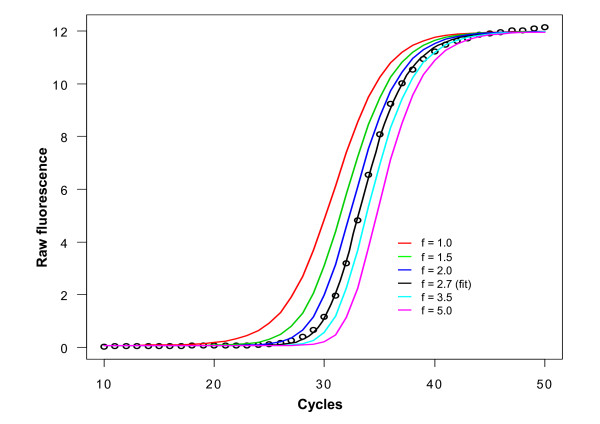
**Effect of different f-parameter values on a fitted qPCR curve**. A five parameter log-logistic model was fitted on the real-time PCR curve of the S27a transcript (black line, fit; black circles, experimental measurements). The effect of varying the f-parameter between 1.0 and 5.0 while keeping the other parameters constant is displayed as coloured curves. Altering the f-parameter also influences other parameters of the fit, mainly 'b' (the slope) and 'e' (the inflection point), although the latter is in a five parameter model different from the classical definition (see Discussion).

Asymmetry of the lower and upper part of the sigmoidal curve (in respect to the inflection point) is the main feature that discriminates the five-parameter models from the four-parameter models. The asymmetry is governed by the fifth parameter which we will denote f in the sequel. The f-parameter has a strong effect on the general fit which is exemplified by altering the f-parameter of a perfect five-parameter sigmoidal fit while keeping all other parameters constant (Figure 1). Changing the f-parameter also influences to a high degree the parameters b (slope) and e, which is a consequence of all parameters being in the denominator of the five-parameter function (Equations 1 and 2). As a consequence of this, the non-linear fitting procedure does not only add a fifth parameter to the four-parameter model, but also adjusts all other parameters to minimize the sum of squared residuals, such that the two models are not directly comparable from the resulting parameter values; for example the parameter e is no longer the inflection point of the sigmoidal curve when f ≠ 1.

**Figure 2:**
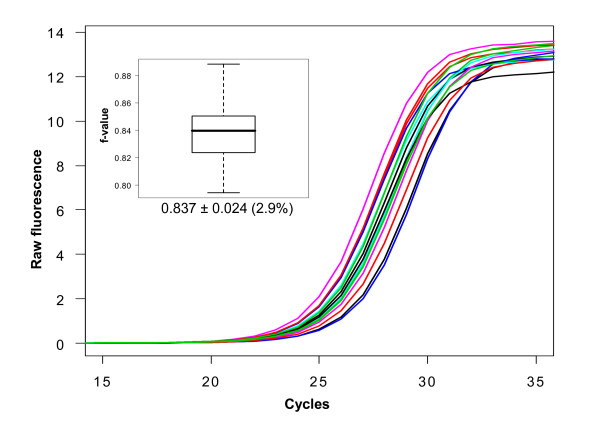
**Scenario A: qPCR curves with different threshold cycles can have similar f-values**. PCR amplification of the transcript of the tumor necrosis factor (TNF) was conducted on 15 different RNA samples obtained from human testicular tissue. The threshold cycles varied within a range of 3 cycles (24.2 to 26.8). The inset shows the boxplot of the f-values from 15 different amplification curves.

**Figure 3:**
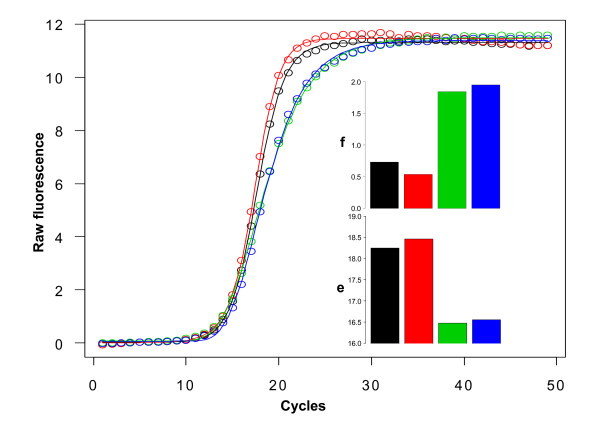
**Scenario B: qPCR curves with similar threshold cycles can have different f-values**. PCR amplification of the S27a transcript on the same RNA in two different duplicates, with the second duplicate set (blue and green line) obtained with only 70% of the polymerase concentration as in the first set (red and black line). The threshold cycle obtained from the second derivative maximum method was within 13.6 ± 0.3 cycles. Both insets depict the parameter estimates for 'f' and 'e' for the fitted curves, showing that these two parameters are highly correlated. Black circles indicate experimental measurements.

The estimation of the f-parameter in two different classical qPCR scenarios was investigated. The first experiment (Scenario A), obtained from 15 qPCR runs with slightly differing starting template amounts, resulted in raw fluorescence data with similar curvature but different threshold cycles (Figure 2). Although these varied within a window of 3 cycles (which would be equivalent to a difference in starting template of 8-fold, simplifying an efficiency of 2), the f-parameter stayed constant at a value of 0.837 ± 2.9%. A different dataset (Scenario B) from two qPCR runs with slightly differing amounts of *Taq *polymerase, featuring exactly the same threshold cycles (13.6 ± 0.3 cycles) but different curvature, showed very different f-parameters. An effect of the f-parameter on the e-parameter (Figure 3) is also evident. The values for e do not define the point of inflection as is the case in four-parameter models. Thus, one must be cautious to compare the two models in respect to the obtained parameters.

### Model selection for the best fit and statistical analysis

**Figure 4:**
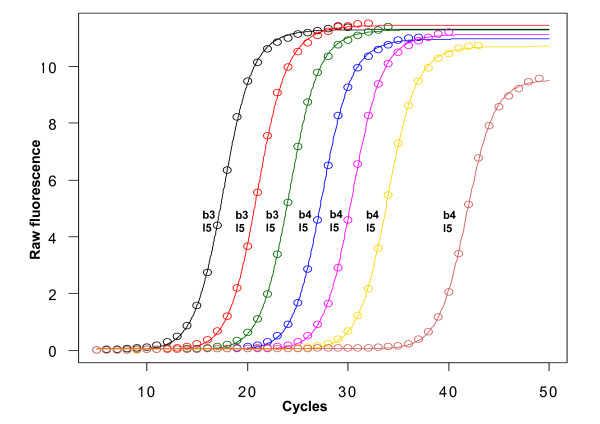
**Model selection of logistic fits on a qPCR dilution experiment**. Three-, four- and five-parameter logistic ('b3'-'b5') and log-logistic ('l3'-'l5') models were fit onto the average of quadruplicate S27a amplification curves of a 10-fold dilution experiment. The best models among the logistic models and log-logistic models were selected by nested F-tests using the residual variance as the criterion and are displayed in black letters near the curves. Coloured circles indicate experimental measurements.

In order to compare the five-parameter models with their according four- or three-parameter versions, a proper statistical measure for the goodness-of-fit of two different models has to be established. The most common method for comparing models that are nested is the F-test based on the residual variance from the fit [18], which is also the method we applied in our context and is the basis of analysis within the *qpcR *package. This gives rise to comparison of models within a nested regime, i.e. the same basic formula but with an added parameter. The F-test was used for the investigation of optimized fits in the analysis of PCR data, comparing either the five-parameter logistic function with its four-parameter counterpart (in short: 'b5' with 'b4'), or the according log-logistic functions (in short: 'l5' with 'l4'). The best model is selected when the F-test between two successive models results in p < 0.05. A typical outcome of the model selection process with a serial dilution experiment (seven 10-fold dilutions, with four replicates each) is depicted in Figure 4, with the tendency to show significantly better fits for the 'b4' model than the 'b3' model in PCR data with higher threshold cycles, i.e. higher dilutions. The asymmetric five-parameter log-logistic model ('l5') performs best irrespective of the threshold cycle and throughout all curves. To validate the performance of the model selection process on a different platform, four independent serial dilution data sets were considered. The datasets differed in the number of replicates, platform and enzymatic chemistry (see Additional file 1). Significantly better fits of the five-parameter model over the four-parameter model, as measured by nested F-test, occurred in 16 of 24 replicates (Dataset 1), 30 of 30 (Dataset 2), 19 of 21 (Dataset 3) and 47 of 48 (Dataset 4).

The application of the model selection process is based on the F-test significance from the fit of the complete (or the largest part) of the amplification curve. As the PCR efficiency and second derivative maximum are derived mainly within the exponential region, it was necessary to evaluate the performance of the five-parameter model with a measure for the goodness-of-fit solely within this important part of the amplification curve. We identified the exponential region by two different methods: (i) the studentized residuals method as described in [9] and (ii) by fitting an exponential model with a window of seven points along the complete amplification curve and identifying the region with the smallest residual variance of the fit. The outcome from both methods was nearly always identical.

**Figure 5:**
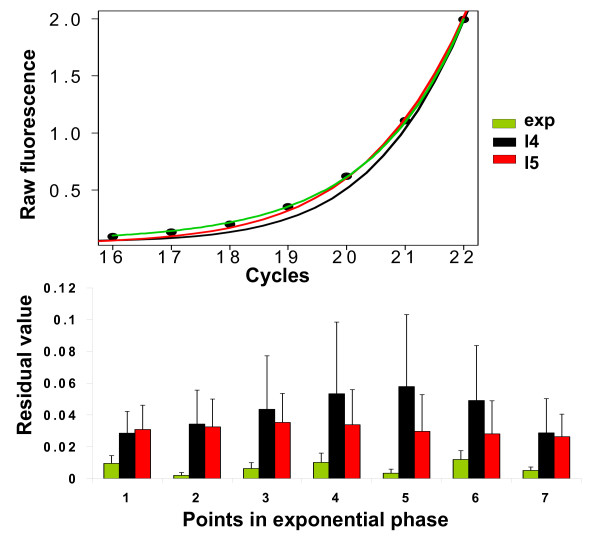
**Goodness-of-fit in the exponential region of the amplification curve**. Upper panel: Zoom-in view for the goodness of fit in the exponential region of a qPCR curve. Three different models were fit to the exponential region of a typical qPCR curve which was identified by the method described in [9]: An exponential model (green), a four-parameter log-logistic model (black) and a five-parameter log-logistic model (red). Lower panel: Residual values for seven points within the exponential regions of 24 qPCR data fits (six dilutions, four replicates each) for the three models. Colour coding same as above. Values are shown as mean + s.d.).

The performance of the 'l5' model in comparison to the 'l4' model and the exponential model is shown in Figure 5 (upper panel). The best fit is exhibited by the exponential model, while the five-parameter log-logistic model clearly outperforms its four-parameter counterpart. To confirm this observation, we calculated the residual value (i.e. observed value minus fitted value) for each of the seven points within the exponential phase for 24 different PCR runs in different dilutions (Figure 5, lower panel). Corroborating the findings above, the exponential fit has the best performance (and least variation), followed by the five-parameter model. By focussing only on the goodness of fit within the exponential region of the qPCR data, increased performance for the five-parameter model over its four-parameter counterpart was observed in 9 of 24 cases (Dataset 1), 30 of 30 (Dataset 2), 21 of 21 (Dataset 3) and 47 of 48 (Dataset 4). Interestingly, for some replicates of Dataset 3 & 4 the five-parameter model outperformed even the exponential model in respect to the root-mean-squared-error (12/21 and 21/48, respectively).

### Estimation of essential qPCR parameters from the five-parameter model compared to previously established quantification models

For relative quantification of qPCR data, the estimates of the PCR efficiency have to be combined with the results from the threshold cycle analysis. Thus, it was necessary to derive the five-parameter equivalent of the threshold cycle, which is implemented in the *qpcR *package as the second derivative maximum (cpD2). The efficiency is then estimated at this point (see Equation 8). The calculation of the parameters follow the model selection step, such that they are based on the best performing sigmoidal model.

**Figure 6:**
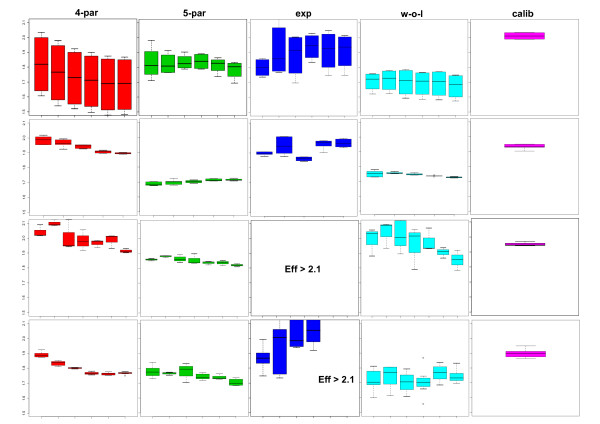
**Assessment of quantitative real-time PCR efficiencies from the replicates of four independent dilution datasets**. Four independent datasets (in rows) were analyzed in respect to the PCR efficiencies of five different methods (in columns), as follows: 4-par: a four-parametric log-logistic model; 5-par: a five-parameter log-logistic model; exp: an exponential model after outlier cycle detection [9]; w-o-l: the window-of-linearity method as described in [8]; calib: a calibration curve obtained from linear regression of all threshold cycles. The boxplots depict the statistical features of the replicates within each dilution step. For methods 1–4, efficiencies were calculated per curve, while for method 5 one efficiency estimation was obtained from all dilution steps. If estimated efficiencies were larger than 2.1, this is denoted in the graphs. The derivation of the efficiency from four- and five-parameter models was done with Equation 8.

The estimation of the second derivative maximum was found to be more reproducible with the five-parameter model in two of four datasets (Dataset 2 and 3). In respect to the PCR efficiency, Datasets 1–3 showed a higher reproducibility with the five-parameter method that was also prominent within the efficiency estimates obtained from other methods (Figure 6). The calibration curve method has the highest reproducibility, which underlines its status of being the 'gold standard' but the reproducibility of efficiency estimates based on the five-parameter model comes close. An additional observation is that within the different serial dilution steps, the efficiency estimates are spread more evenly around a fixed value, i.e. the variation of efficiency values within a dilution regime is much smaller.

### Accuracy and precision of ratio estimates obtained from Δct methods and initial template fluorescence (F0)

In general, two methods exist for relative quantification of qPCR data. Ratios based on threshold cycles and efficiency estimates are most commonly applied when using single curve methods such as the four-parameter model, the exponential model and the 'window-of linearity' method. Furthermore, these methods can estimate the initial template fluorescence at the beginning of the curve, often termed F0. Although it has been repeatedly claimed that a fair estimate of F0 can be obtained by setting x = 0 in the sigmoidal function [10,17], we failed in getting reproducible data from this approach with all datasets (not shown). Instead, we calculated F0 by using an exponential model with the parameters estimated from the five-parameter fit (Equation 9). We evaluated the four dilution datasets using the Δct and F0 values for the five methods, where applicable, and calculated the accuracy and precision of the estimated dilution ratios (Table 1).

**Table 1:** Summary for accuracy and precision of dilution ratio quantitation obtained from four independent dilution datasets.

	**Set 1**	**Set 2**	**Set 3**	**Set 4**
**sigm/Δct/4-par**	65.5 (36.9)	106.4 (7.0)	108.3 (16.6)	84.9 (7.6)
**sigm/Δct/5-par**	**73.5 (21.9)**	77.2 (**4.5**)	89.3 (**4.6**)	75.9 (8.3)
**sigm/F0/4-par**	46.6 (35.8)	79.7 (15.2)	117.3 (79.1)	68.8 (21.1)
**sigm/F0/5-par**	**70.4 **(42.4)	**86.1 **(17.8)	**87.1 **(33.6)	**72.6 **(48.6)
**exp/Δct/4-par**	85.7 (21.8)	104.2 (11.2)	318.8 (31.7)	339.5 (60.7)
**exp/Δct/5-par**	82.6 (21.2)	**101.3 (10.2)**	319.74 (29.9)	355.4 (61.3)
**exp/F0**	198.0 (93.1)	238.9 (92.6)	79.2 (134.0)	N.V.
**w-o-l/Δct/4-par**	59.4 (18.2)	83.9 (5.4)	105.7 (19.5)	74.1 (20.7)
**w-o-l/Δct/5-par**	57.5 (18.3)	81.9 (5.0)	**103.9 (14.9)**	73.9 (21.8)
**w-o-l/F0**	53.5 (25.9)	76.3 (14.3)	310.2 (118.2)	295.8 (175.6)
**calib/Δct/4-par**	126.0 (21.6)	100.6 (5.8)	100.6 (11.5)	99.9 (7.7)
**calib/Δct/5-par**	128.6 (20.8)	97.9 (5.1)	99.3 (5.9)	99.3 (8.1)

Five commonly used quantification methods in conjunction with threshold cycles estimated from four- and five-parameter sigmoidal models were applied for the analysis. Four different datasets differing in the number of replicates, enzymatic chemistry and platform were analyzed in respect to accuracy (average percentage of calculated ratios from real ratios) and precision (average c.v.; numbers in brackets). Threshold cycles estimated from the second derivatives maximum of four- and five-parameter sigmoidal models (4-par, 5-par) were used in combination with the following methods: sigmoidal model with Δct method (sigm/Δct), exponential model with Δct method (exp/Δct), window-of-linearity method with Δct method (w-o-l/Δct) and calibration curve with Δct method (calib/Δct). Methods for calculation of ratios based on the initial template fluorescence (F0) were: four-parametric sigmoidal model (sigm/F0/4-par), five-parameter sigmoidal model (sigm/F0/5-par), exponential model (exp/F0) and the 'window-of-linearity' method (w-o-l/F0).

Numbers in bold are combinations in which the five-parameter model performs best. N.V. : no realistic estimation values due to problematic fits.

The performance of using the Δct method with efficiency and threshold cycles estimated from the five-parameter model is increased in three datasets but is found within different methods. In contrast to this observation, ratios estimated by initial fluorescence from the five-parameter sigmoidal fit (sigm/F0/5-par) presented higher accuracy and precision throughout all datasets.

## Discussion and Conclusion

By fitting four-parameter sigmoidal models onto many datasets, we observed that the fitted curves were often not optimal at the ground asymptote, top asymptote ('plateau phase') and even more important at the log-linear region that is used for the estimation of PCR efficiencies or threshold cycles. As an asymmetrical structure of the data would be a proper explanation for this phenomenon, we analyzed the performance of fitting five-parameter models onto qPCR data.

The fifth parameter (termed 'f' in this work) has profound impact on the sigmoidal curvature of the fit. When equal to 1, the five-parameter fit is reduced to its four-parameter equivalent. We rarely observed values very near to 1 after non-linear fitting. This is the reason why we emphasize the use of the five-parameter models, since asymmetry of qPCR data seems to be an inherent characteristic and absolutely symmetric qPCR data seldom (in our observations never) occur. As shown on different qPCR scenarios, the asymmetry parameter is unique to every curve and due to its interaction with other parameters of the fit (mostly 'e', the inflection point and 'b', the slope) the results of the fit are often similar but not directly comparable to four-parameter models.

To base our new proposed model on solid statistical ground, we conducted a nested F-test of the new five-parameter models versus the four-parameter versions in order to validate the increased performance. This is common practice for selecting the best model in non-linear fitting regimes [18] and delivers the essential p-value for choosing the fit with the smallest residual variance. Statistical significance in the region of p = 10^-3 ^to p = 10^-16 ^of five-parameter logistic or log-logistic models over their four-parameter counterparts were seen in almost all qPCR curves we examined. The log-logistic model 'l5' has the highest occurrence within the model selection, but we also observed fits with the logistic 'b5' model performing best, especially when the raw fluorescence data has low values. We believe that the advantage of the log-logistic model over the logistic model is in a reduced effect of the plateau cycles on the fit as a consequence of the logarithmized x-values (cycle numbers).

By using the RMSE and statistics based on the residual values, it could be shown that the five-parameter models clearly outperform their four-parameter counterparts in fitting the model solely to the exponential region. In most of the cases the performance of the exponential fit, which exhibited very low RMSE values and highly accurate fitting characteristics, was superior. This was not the observation for the dataset from Rutledge *et al*. [17] and another dataset from our group, where the five-parameter log-logistic model surpassed the exponential model. These two datasets exhibit lower raw fluorescence values in general, such that the reason for the different performances is likely to be based on the underlying platform or enzymatic system. The exponential model does not fit optimal on this kind of data and the fitting procedure was often problematic and yielded unsatisfactory estimates.

The reproducibility of the efficiency estimation with the five-parameter models was not only significantly better than with the four-parameter models, but also often surpassed the reproducibility of the exponential model and the 'window-of-linearity' method. This characteristic was found for the same datasets with low exponential fitting performance as described above.

In the aforementioned work from authors utilizing four-parameter models, the feasibility of using these were corroborated by using the R^2^-value as the figure-of-merit, demonstrating very high values (R^2^>0.99). As we have seen, the R^2^-value is not a sensitive measure for model comparison: Dramatic improvement (as is often the case when going from symmetry to asymmetry) is hardly being reflected in the R^2^-value. There is considerable controversy about the use of this measure in non-linear fitting [19]. Consequently, we would like to advocate that this measure should not be reported or trusted solely for demonstrating the validity of a fit in sigmoidal qPCR data.

The introduction of the five-parameter model is in our opinion another leap in the direction of automatic qPCR data analysis. This intention, introduced in [17] is a project to be still reached. It is unfortunately a fact that different methods in qPCR analysis can yield very different values in respect to PCR efficiency, threshold cycle or estimation of the exponential phase [20,21]. Yet, when focusing the attention on sigmoidal models, we believe that the additional aspect of asymmetry is an important feature to take into consideration, since the performance of the fit in each part of the curve (and most importantly in the exponential region) is nearly always improved by using five-parameter models.

## Methods

### RNA extraction and cDNA synthesis

Total RNA was extracted from human testicular biopsies with RNApure™ (Peqlab, Germany) and re-purified on RNeasy™ columns (Qiagen, Germany) according to the manufacturers' protocols. RNA purity and integrity (28S/18S ratio) were assessed by loading aliquots of approximately 200 ng onto RNA 6000 nano assay chips using an Agilent Bioanalyzer (Model 2100; Agilent Technologies, Palo Alto, CA). Only samples with an RNA integrity number higher than 7.5 (RIN, Agilent software) were included for the PCR experiments. cDNAs were synthesized with Superscript™ II reverse transcriptase (Invitrogen, Carlsbad, CA) according to the manufacturers' protocol.

### Quantitative real-time PCR (qRT-PCR)

qRT-PCR was performed using LightCycler™ (Roche, Basel, Switzerland) technology using 10 pmol each gene specific primers, 2 μl dNTP mix (25 mM each, Takara Bio, Shiga, Japan), 0.5 μl SybrGreen I (1:1000 in DMSO; Molecular Probes, Leiden, Netherlands), 0.25 μl BSA (20 mg/ml; Sigma, Germany) and 0.2 μl Ex-Taq HS (5 U/μl; Takara Bio, Shiga, Japan) in a total volume of 20 μl. Cycling conditions were 95°C 5 min, 95°C for 10 s, 60°C 10 s, 72°C for 30 s with a single fluorescence measurement at the end of the segment, repeated for 50 times. A melting curve program (60–95°C with a heating rate of 0.1°C/s and continuous fluorescence measurement) was conducted and the PCR products were electrophoretically separated on 1.3% agarose/TAE gels and verified by sequence analysis.

### Curve fitting

Both the curve fitting process and the data analysis were conducted with the *qpcR *package, which is tailored to the special application of real-time polymerase chain reaction and houses several functions for fitting different curve types to qPCR data. The fitting process and model selection is done by using functionality from the package *drc *[14], which is the statistical analysis engine, while the *qpcR *package extends the fitting process by deriving several important qPCR parameters, providing optimization procedures and graphical evaluation of the results. All raw data used for the analysis were not processed (i.e. baseline corrected).

We compared the most widely applied sigmoidal curve type for qPCR analysis, the four-parameter logistic curve (also termed *Boltzmann *fit) and additionally its four-parameter log-logistic counterpart to five-parameter versions that were shown to exhibit better fits for asymmetric data [15].

The applied four- and five-parameter sigmoidal curves are related by the following equation (x is the cycle number):

(1) $$\begin{array}{cc} \text{g(x, b, c, d, e,f)}=\text{c}+\frac{\text{d}-\text{c}}{{\left(\text{1}+{\text{e}}^{\text{b(log(x)}-\text{log(e))}}\right)}^{\text{f}}} & \text{five-parameter log-logistic curve} \end{array}$$

(2) $$\begin{array}{cc} \text{g(x, b, c, d, e,f) }\text{​}=\text{c}+\frac{\text{d}-\text{c}}{{\left(\text{1}+{\text{e}}^{\text{(b(x}-\text{e))}}\right)}^{\text{f}}} & \text{five-parameter logistic curve} \end{array}$$

The parameters b, c, d and e correspond to the slope, ground asymptote, maximum asymptote and the inflection point, respectively. The parameter f is the additional asymmetry parameter. Setting parameter f = 1 yields the four-parameter curves previously described. The parameters of the four- and five-parameter fits are estimated by method of non-linear least squares which seeks to find the parameters that minimize the residual sum-of-squares (y is the raw fluorescence measurement):

(3) $$\sum_{i=1}^{n}{\left(y_{i}-g(x_{i},b,c,d,e,f)\right)}^{2}$$

with respect to the parameters (b, c, d, e) and also f in case of the five-parameter models. This procedure is in principle feasible with any data analysis software that is capable of fitting non-linear regression models, but is much more conveniently accessible through built-in models (such that the user need not provide initial values ("guesses") of the parameter values). The four-parameter models are commonly available in various data analysis software. Tools exclusively for the five parameter models are not generally available, two exceptions being the StatLIA software (Brendan Technologies) and the five-parameter log-logistic Richards model found in GraphPad Prism version 5.0 (Graphpad Software Inc...).

### Choosing the best model by nested F-tests

Finally, after fitting the different sigmoidal models to the data, a model selection process is conducted to find the model that exhibits a best fit in respect to the sum-of-squares. One approach, which is only valid when the models are nested (i.e. an additional parameter on the same basic formula is applied) is to use an F-test, often called an extra-sum-of-squares test. The F-test quantifies the relative decrease in sum-of-squares when going from a simpler model to a more general model:

(4) $$F=\frac{\left(rss_{4pl}-rss_{5pl}\right)/\left(df_{4pl}-df_{5pl}\right)}{rss_{5pl}/df_{5pl}}$$

with rss = residual sum-of-squares = $\sum_{i=1}^{n}{\left(y_{i}-{\hat{y}}_{i}\right)}^{2}$ (y_i _= the actual y value, ${\hat{y}}_{i}$ = the fitted value), df = degrees of freedom, 4pl = four-parameter fit and 5pl = five-parameter fit. The p-value obtained from an F-distribution then evaluates the chance that -if the experiment were repeated- one randomly obtains data that would yield an even larger relative decrease than observed for the actual data.

### Evaluating the different measures of goodness-of-fit for sigmoidal models

The *qpcR *package can also employ the Akaike Information Criterion (AIC) as an additional figure-of-merit which is applicable for comparing different models that are not necessarily nested [16]. Increasing the number of parameters to be estimated always improves the goodness of the fit, but at the cost of increased uncertainty as more parameters are estimated with a constant amount of information (the same data). The AIC rewards the improvement in the fit, but to reflect the increased uncertainty AIC also includes a penalty that is an increasing function of the number of parameters:

(5) $$AIC=2k+n\cdot \ln\left(\frac{rss}{n}\right)$$

with rss = residual sum-of-squares, k = number of parameters (i.e. k = 5 in a five-parameter model) and n = number of observations. The preferred model is the one with the lowest AIC value. A related measure of the quality of a model fit is the residual variance which is defined as:

(6) $$resVar=\frac{rss}{n-k}$$

with rss, n and k as previously defined.

An often used measure for the goodness-of-fit is the regression coefficient, R^2^:

(7) $$R^{2}=1-\frac{rss}{tss}$$

with rss = residual sum-of-squares and tss = total sum-of-squares = $\sum_{i=1}^{n}{\left(y_{i}-\bar{y}\right)}^{2}$ (where $\bar{y}$ is the average of the *y *values). This measure has been used to demonstrate the validity and goodness of four-parameter sigmoidal models in the analysis of real-time PCR data [10,11,17].

### Deriving essential PCR parameters from the five-parameter models

For the quantification of real-time PCR experiments, the two parameters 'PCR efficiency' and 'threshold cycle' (also referred to as 'crossing point') are essential in the calculation of relative differences of DNA abundance. Within the *qpcR *package, the efficiency can be calculated along any point of the sigmoidal fit, but is taken at default at the second-derivative maximum. In most of the cases, this point lies within the exponential region of the curve. Thus, by defining Eff = F(x)/F(x-1), follows

(8) $$EFF_{cpD2}=\frac{F(cpD2)}{F(cpD2-1)}$$

with F = raw fluorescence at cycle x, and cpD2 = cycle number at second derivative maximum of the curve.

The calculation of the initial template fluorescence F0 for the exponential model and the 'window-of-linearity' method has been published elsewhere [11,8]. For sigmoidal models, two methods for estimation of F0 exist: Calculation of F0 by setting x = 0 in the sigmoidal function (Equations 1 & 2) or by extrapolating an exponential model using the parameters estimated from the (four- or five-parameter) sigmoidal fit:

(9) $$F0=\frac{F(cpD2)}{\left(Eff_{cpD2}\right){\;}^{cpD2}}$$

with F(cpD2) = raw fluorescence at second derivative maximum cycle number, Eff_cpD2 _= efficiency at second derivative maximum as calculated by Equation 8 and cpD2 = second derivative maximum cycle number.

Estimates for the PCR efficiency, the maxima of the first and the second derivatives and the initial template fluorescence based on the five-parameter sigmoidal fits are derived within the *qpcR *package and were used for calculations of four independent dilution datasets.

### Comparison of the new model to previously established models

We compared the five-parameter models with previously established models such as the four-parameter sigmoidal model [10], the window-of-linearity method [8], exponential fitting after identification of outlier cycles by studentized residuals [9] and from the slope of a calibration curve [5]. Reproducibility for all essential parameters of each method were calculated from replicate experiments of four independent dilution datasets that differ in the number of replicates, platform and enzymatic chemistry (see Additional file 1). To evaluate the performance of these methods in respect to accuracy, we calculated the average percentage of the estimated dilution ratios compared to the technical dilution ratios:

(10) $$Accuracy\left[\%\right]=\frac{\text{mean of estimated dilution ratios}}{\text{technical dilution ratio }}\cdot 100]$$

As the most important information for comparison of the non-linear models is the performance of the fit in the exponential region of the amplification curve, we analysed the goodness-of-fit in this region by the commonly used root-mean-squared-error (RMSE):

(11) $$RMSE=\sqrt{\frac{{\sum res}^{2}}{n}}$$

with res (residual) = actual value – predicted value (fit) and n = number of points. One can compare different models by absolute comparison of the RMSE values for the different models: the smaller the RMSE the better the model fit.

## Authors' contributions

ANS analyzed the data, developed the *qpcR *package and drafted the manuscript. CF conducted the quantitative real-time PCR experiments. CR coded the five-parameter model and the model selection process.

## Supplementary Material

Additional file 1 **Statistical summary of four independent dilution datasets and five commonly used quantification methods based on threshold cycles and initial template fluorescence as estimated from four- and five-parameter sigmoidal models**. Four different dilution datasets differing in the number of replicates, enzymatic chemistry and platform were analyzed in respect to efficiency, measures for the goodness of fit (RMSE, AIC, R-squared; see Methods), threshold cycles estimated from four- and five-parameter sigmoidal models and calculated ratios obtained either by Δct methods or by estimation of the initial template fluorescence (F0). In total, five different methods as described in the file under 'Details' were used for the statistical comparison (see also Legend to Table 1). In cases of increased performance of the five-parameter models over the four-parameter models, the statistical values were highlighted in yellow. Microsoft Excel spreadsheet. Click here for file
